# MMSE in primary care practice: why good tests can mislead in the wrong context

**DOI:** 10.1007/s10433-025-00894-6

**Published:** 2025-11-04

**Authors:** Carla Tortora, Laetitia Teixeira, Susana Sousa, Constança Paúl

**Affiliations:** 1 Department of Psychology, University “G. d’Annunzio” of Chieti-Pescara, Via Dei Vestini 31, Chieti, Italy; 2 University of Porto, Porto, Portugal; 3 RISE - Health, Porto, Portugal

**Keywords:** Primary care, Cognitive assessment, Dementia, Neuropsychology, General practitioners

## Abstract

The Mini-Mental State Examination (MMSE) is widely used in cognitive screening, including in primary care settings. This study evaluated the diagnostic performance of the MMSE using data from 390 community-dwelling older adults aged 65 to 98 years (*M* = 75.76, SD = 6.76). The MMSE’s accuracy was assessed against clinical diagnoses and symptom severity levels based on the Global Deterioration Scale (GDS). Receiver operating characteristic (ROC) analysis, evaluating the ability to distinguish individuals with dementia from those without, provided an area under the curve (AUC) of .75 (95% CI: 0.67–0.84, *p* < .001). The optimal cutoff based on Youden Index was 21 and resulted in a sensitivity of .77 (95% CI [.728, .812]) and specificity of .65 (95% CI [.603, .697]), whereas the more conventional cutoff (24) showed lower sensitivity (.50; 95% CI [.450, .550]) but higher specificity (.82; 95% CI [.782, .858]). At the suggested cutoff, the MMSE identified all cases at the severe stage, 88% at the moderate stage, and 31% at the mild stage of dementias, as classified by the GDS. In contrast, the Quick Mild Cognitive Impairment screen (Qmci) identified nearly all cases across severity levels. Against previous dementia diagnoses, when employing a cutoff score of 24 the MMSE had a positive predictive value of .52 (95% CI [.395, .645]) and a negative predictive value of .81 (95% CI [.726, .894]), indicating modest diagnostic reliability in a primary care context. Similar results were obtained applying a cutoff score of 21. These findings highlight how base rates and test characteristics shape test accuracy and should guide decision-making. Overall, our findings highlight that the MMSE can produce a substantial number of false positives in contexts with a relatively low prevalence of dementia, such as primary care, challenging the common assumption of its low false-positive rate. More broadly, our study emphasizes the importance of considering the prevalence of the condition in a given context, as differences in prevalence can drastically affect the interpretation of results, particularly the positive predictive value, even when sensitivity and specificity remain unaffected.

## Introduction

The Mini-Mental State Examination (MMSE; Folstein et al. 1975) remains one of the most widely used tools for cognitive assessment and is still often considered a gold standard in clinical practice. Due to its brevity and ease of administration, it is frequently the first-choice instrument for cognitive screening, including in primary care settings. While its diagnostic performance has been shown to be relatively high in specialized clinics and secondary care settings, several studies have suggested that its accuracy is significantly reduced when applied in primary care contexts (Janssen et al. 2017; Arevalo-Rodriguez et al. 2021; Karimi et al. 2022). Despite substantial efforts in literature to propose more comprehensive and psychometrically robust protocols tailored to the primary care context (Janssen et al. 2017), brief global tools such as the MMSE continue to dominate clinical practice (Limongi et al. 2019; Fernandes et al. 2021; Karimi et al. 2022).

**Fig. 1:**
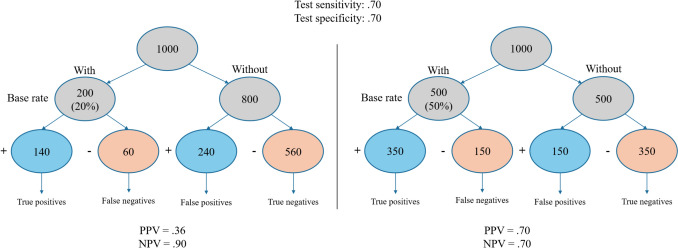
Illustration of predictive values of a test with .70 sensitivity and specificity in a primary care setting based on a hypothetical cohort of 1000 individuals aged 65 + , assuming 20% (left side) and 50% base rate (right side) of cognitive impairment. Note that test sensitivity and specificity were chosen based on previous research on MMSE accuracy in primary care contexts

The main issue with using the MMSE in primary care settings lies in the fact that, regardless of its sensitivity and specificity, its positive and negative predictive values tend to be lower in these contexts due to the lower base rate of cognitive impairment (Creavin et al. 2016; Fischer 2021). The base rate, that is, the actual prevalence of a condition in the population being tested, plays a critical role in shaping the predictive values of a diagnostic tool. Specifically, the positive predictive value (PPV) indicates the likelihood that someone who tests positive truly has the condition, while the negative predictive value (NPV) reflects the likelihood that someone who tests negative truly does not have it. These values are not intrinsic to the test itself but are heavily influenced by how common the condition is in the population. Bayes' theorem provides a mathematical framework to update the probability of a condition based on both the test result and the pre-test probability (or base rate). In primary care, where the base rate of cognitive impairment is relatively low compared to specialized memory clinics (Fiest et al. 2016), even a test with good sensitivity and specificity like the MMSE can produce a high number of false positives and a lower PPV. In contrast, the NPV tends to remain higher in such settings, meaning that negative results are more trustworthy, although still not ideal. An example of how base rate affects both PPV and NPV can be found in Fig. 1. As shown in the example, with a lower base rate (20%) the NPV tends to be higher; however, there would still be a relatively high number of false negatives, which are usually more problematic than false-positive cases as the possibility of further evaluation may be compromised. This statistical limitation is compounded by a clinical one: The MMSE is generally more effective in identifying dementia or more severe symptomatology of cognitive impairment (Liss et al. 2021). However, general practitioners and family doctors are often the first point of contact for individuals experiencing subtle or early cognitive changes. In this role, they are uniquely positioned to promote early screening and timely interventions, when they are most likely to be effective in preserving cognitive functioning and quality of life. Relying on a tool that performs best in detecting more severe cases risks missing early, actionable stages. Nonetheless, clinicians may not always be aware of how base rates influence the predictive value of test scores, potentially leading to misinterpretations and missed opportunities for early intervention (Morgan et al. 2021). Indeed, it is often perceived, though inaccurately, as a safer tool because of its anticipated lower false-positive rate. The underlying bias is that, while it may miss many early cases (i.e., false negatives), a score below the cutoff is generally interpreted as a strong indicator of true dementia. However, in primary care contexts, where the base rate of dementia is relatively low, the interpretation of cognitive test results requires caution.

Despite this, and although these limitations are well documented in literature and sometimes acknowledged by clinicians (Naugle and Kawczak 1989; Arevalo-Rodriguez et al. 2021; Mandyla and Kosmidis 2023), the use of the MMSE in primary care remains widespread, and not without reason. Primary care physicians often face significant barriers to conducting in-depth cognitive assessments, including limited time, insufficient resources, and workforce shortages that leave them responsible for a disproportionately high number of patients (Costa et al. 2024). As a consequence, early detection of cognitive impairment is frequently unfeasible, leading to delayed diagnoses when therapeutic options are more limited and less effective. This delay has profound social implications and contributes to the emotional and financial burden on families, who often provide care for individuals with advanced cognitive impairment in the absence of adequate support systems (Borson et al. 2023). Moreover, these challenges tend to disproportionately affect individuals from lower socioeconomic backgrounds, thereby exacerbating existing health disparities (Villarejo Galende et al. 2021; Wimo et al. 2023). Additionally, the MMSE may continue to be preferred in primary care settings due to physicians having greater familiarity with the instrument and the perception that it is more straightforward to administer and interpret. In some cases, physicians may also be reluctant to adopt more sensitive tools due to concerns about overdiagnosis or the potential stigma associated with labeling patients at early stages of cognitive impairment (Bacsu et al. 2020).

The present study, which represents a secondary objective within a broader research framework, aims to evaluate the diagnostic accuracy of the MMSE in primary care settings. Specifically, it investigates how well the MMSE identifies cognitive impairment compared to other cognitive screening tools and assesses the extent to which its results can be considered trustworthy in real-world primary care conditions.

## Methods

### Participants

The present study included 390 community-dwelling older adults aged between 65 and 98 years (*M* = 75.76, SD = 6.76), of whom 162 (41.5%) were men and 228 (58.5%) were women. Participants had between 0 and 17 years of formal education (*M* = 3.17, SD = 2.39), with the majority (91.3%) having completed 4 years or less.

### Procedure

This study is part of a larger research project conducted in Northern Portugal, which aimed to assess risk factors and health outcomes among community-dwelling older adults with probable dementia (for a more in-depth explanation of the main study methodology please see Teixeira et al. 2017). The original sample was drawn from individuals aged 65 and older, identified as being at risk for mental health concerns by primary care professionals using the Risk Instrument for Screening in the Community (RISC; O’Caoimh et al. 2015). Specifically, the study was conducted in the area covered by the Portuguese North Regional Health Authority (ARS North), which included 86 municipalities organized into 24 Health Centre Associations (ACES). At least two primary care units were recruited from each ACES based on interest in participation, resulting in 55 units and 285 health professionals completing an initial screening for 7298 patients. Patients with probable dementia identified in the screening were randomly sampled, leading to a final sample of 436 patients with probable dementia. The study protocol, based on the “Community Assessment of Risk and Treatment Strategies (CARTS) Program” (O’Caoimh et al. 2012), included three parts: Part A assessed the patient with probable dementia, Part B involved evaluation by the health professional (general practitioner or nurse), and Part C included an assessment of the informal caregiver when available. The study was approved by the Ethics Committee of the ARSN (Opinion no. 6/2014) and was conducted in accordance with the Declaration of Helsinki (2013). From the initial sample, only individuals who completed both the Mini-Mental State Examination (MMSE) and the Quick Mild Cognitive Impairment (Qmci; O’Caoimh and William Molloy 2016) Screen without missing values were included in the present analyses, resulting in a final subsample of 390 participants. These instruments were part of the standardized assessment protocol administered by trained interviewers either in healthcare facilities or at the participants’ homes, depending on their mobility status. Written informed consent was obtained from all participants or their legal representatives prior to participation.

### Instruments

Together with a sociodemographic questionnaire used to collect basic information about the participants (e.g., age, gender, level of education), global cognitive functioning was assessed using the Mini-Mental State Examination (MMSE; Folstein et al. 1975), which is a brief 30-point screening tool widely used to evaluate cognitive impairment across domains such as orientation, attention, memory, language, and visuospatial abilities. Additionally, cognitive functioning was also assessed using the Portuguese version of the Quick Mild Cognitive Impairment screen (Qmci; O’Caoimh and William Molloy 2016, Portuguese version by Dos Santos et al. 2019), which is a brief, standardized cognitive screening tool designed to differentiate normal cognition, Mild Cognitive Impairment (MCI), and dementia through six cognitive domains: orientation, registration, clock drawing, delayed recall, verbal fluency, and logical memory. In both cases, higher scores indicated better cognitive functioning. Finally, symptom severity was evaluated using the Global Deterioration Scale (GDS; Reisberg et al. 1982), a 7-stage rating scale describing the progression of cognitive and functional decline, particularly in dementia. For analytic purposes, GDS scores were grouped into three categories to reflect increasing severity (very mild or mild, moderate, and severe or very severe).

### Statistical analyses

All statistical analyses were performed using SPSS 30. Given the primarily descriptive aim of the study and the focus on distributional data, with a Bayesian approach to positive and negative predictive values (Webb and Sidebotham 2020), no inferential parametric tests were conducted to compare groups and no a priori hypothesis was formulated.

A receiver operating characteristic (ROC) analysis was first conducted to evaluate the sensitivity and specificity of the Mini-Mental State Examination (MMSE) in identifying cases with a pre-existing clinical diagnosis of dementia, as determined by referring physicians (yes/no). Participants with missing or indeterminate diagnostic information were excluded from this analysis. Sensitivity and specificity values were calculated for the conventional MMSE cutoff score of 24, as well as for the optimal cutoff determined using the Youden Index.

Subsequently, participants were categorized according to their level of symptom severity based on scores from the Global Deterioration Scale (GDS). Within each GDS severity group, the proportion of participants scoring below the MMSE threshold was calculated, both using the standard cutoff and the one derived from the Youden Index. This was presented as a series of cross-tabulations. The same procedure was applied to the Quick Mild Cognitive Impairment screen (Qmci), using the recommended cutoff score from the test manual.

Finally, contingency tables were generated to examine the concordance between MMSE classification (based on both the conventional and Youden-derived cutoffs) and the presence or absence of a previous diagnosis of dementia. Participants without explicit diagnostic information were excluded from this analysis. These data allowed for the calculation of positive predictive value (PPV) and negative predictive value (NPV) of the MMSE in our sample, interpreted within a Bayesian framework and based on observed test performance in this specific population. Confidence intervals (95% CI) for sensitivity, specificity, positive predictive value (PPV), and negative predictive value (NPV) were computed using the Wald method, i.e., $p \pm 1.96*\sqrt {\left( {1 - p} \right)/n}$, where “*p*” represents the estimated proportion and “*n*” the corresponding denominator.

## Results

ROC analysis revealed that the MMSE had an AUC of 0.75 (95% CI: 0.67–0.84, *p* < 0.001). The optimal cutoff, based on the Youden Index, was 21, which was associated with a sensitivity of 0.77 (95% CI [0.728, 0.812]) and a specificity of 0.65 (95% CI [0.603, 0.697]). In comparison, the conventional cutoff of 24 resulted in lower sensitivity (0.50; 95% CI [0.450, 0.550]) but higher specificity (0.82; 95% CI [0.782, 0.858]).

Results of the comparison between different severity stages based on the GDS and diagnostic tools (MMSE and Qmci) can be found in Table 1. Employing the suggested cutoff (24), MMSE identified all cases at the severe stage (*N* = 14), 88% at the moderate stage (*N* = 66), and 31% at the mild stage (*N* = 96). On the other hand, Qmci identified all cases at the severe and moderate stages (*N* = 14 and *N* = 75 respectively) and 93% at the mild stage (*N* = 281). Additionally, the diagnostic accuracy of the MMSE against the previous diagnosis of dementia was evaluated (Table 2). In our sample, the base rate of previously diagnosed dementia was approximately 34%. Using a cutoff score of 24, the MMSE correctly identified approximately 70% and failed to detect cognitive impairment in about 30% of cases. Specifically, the test correctly identified 37 true positives (out of 53) and 67 true negatives (out of 101), with 34 false positives and 16 false negatives. Based on these values, the positive predictive value (PPV) was 0.52 (95% CI [0.395, 0.645]), indicating that 52% of participants with a positive MMSE result actually had dementia according to previous diagnosis. The negative predictive value (NPV) was 0.81 (95% CI [0.726, 0.894]), suggesting that 81% of those with a negative MMSE result were correctly identified as not having dementia based on previous diagnosis. When applying a more conservative cutoff score of 21, the MMSE correctly identified cognitive impairment in approximately 57% of the cases. Particularly, the test identified 30 true positives (out of 53) and 83 true negatives (out of 101), with 18 false positives and 23 false negatives. Thus, in this case, the PPV was 0.62 (95% CI [0.483, 0.757]) and the NPV was 0.78 (95% CI [0.701, 0.859]). Notably, a logistic regression analysis was conducted to examine whether MMSE score, age, and education predicted diagnostic status. The overall model was significant (*χ*^2^(3) = 32.46; *p* < 0.001), and explained 28% of the variance (Nagelkerke *R*^2^ = 0.28). The model correctly classified 75.2% of participants. When entered simultaneously, only the MMSE score was a significant predictor (*B* = 0.15; S.E. = 0.05; Wald = 8.78; *p* = 0.003; OR = 1.159; 95% CI [1.051, 1.278]). Age (*p* = 0.934; OR = 0.998; 95% CI [0.942, 1.057]) and education (*p* = 0.066; OR = 1.262; 95% CI [0.985, 1.617]) were not significant predictors. This suggests that in our sample, age and education did not provide additional predictive value beyond MMSE performance.

**Table 1:** Distribution of cognitive impairment according to the Qmci and MMSE, based on GDS-defined symptom severity (mild, moderate, severe)

		Symptom severity			
		Mild	Moderate	Severe	Tot
Qmci	No	21	0	0	21
	Yes	280	75	14	369
	Tot	301	75	14	390
MMSE (cutoff 24)	No	205	9	0	215
	Yes	96	66	14	176
	Tot	301	75	14	390
MMSE (cutoff 21)	No	259	20	0	279
	Yes	42	55	14	111
	Tot	301	75	14	390

**Table 2:** Comparison between MMSE categorization and previous diagnosis of dementia

		Previous diagnosis		
		Yes	No	Tot
MMSE (cutoff 24)	No	16	67	83
	Yes	37	34	71
	Tot	53	101	154
MMSE (cutoff 21)	No	23	83	106
	Yes	30	18	48
	Tot	53	101	154

## Discussion

The aim of this study was to assess the diagnostic performance of the Mini-Mental State Examination (MMSE) in detecting cognitive impairment in individuals recruited in primary care settings, with particular attention to its positive predictive value (PPV) and negative predictive value (NPV). Given the MMSE’s widespread use and the ongoing reliance on it by general practitioners (Mitchell 2012; Limongi et al. 2019; Tran et al. 2022), especially when time and resources are limited, this study sought to clarify how well the MMSE functions in clinical contexts, and how its utility may be affected by factors such as symptom severity and base rates of cognitive impairment. Our results indicate that the MMSE performs reasonably well in identifying severe symptoms of cognitive impairment, detecting all cases classified as severe based on the Global Deterioration Scale (GDS). Its performance declines for moderate symptoms (88% detection rate) and drops significantly for mild symptoms, where only 31% of cases were identified. In contrast, the Qmci demonstrated markedly higher sensitivity across all levels of symptom severity, including mild cases (93% detection). When evaluated against a prior dementia diagnosis, the MMSE showed a sensitivity of 70% and specificity of 66%, resulting in a positive predictive value (PPV) of 0.52 and a negative predictive value (NPV) of 0.81. Receiver operating characteristic (ROC) curve analysis further confirmed that the MMSE has moderate diagnostic utility, with an area under the curve (AUC) of 0.76. Notably, Youden’s index suggested that an alternative cutoff of 21 (as opposed to the standard 24) improved sensitivity (0.78) but lowered specificity (0.65), reflecting the complex trade-off between false positives and false negatives in clinical decision-making.

Notably, although previous diagnosis of dementia was self-reported by physicians without much control over how it was achieved and with little knowledge about missing values, the sensitivity and specificity of the MMSE in our study are similar to and coherent with results from previous studies on the accuracy of the MMSE in primary care in detecting cognitive impairment (Karimi et al. 2022). This contextual consistency supports the relevance of our findings and allows us to explore an important statistical implication: In context in which the base rate of cognitive impairment is relatively low (e.g., 30%), even a test with balanced sensitivity and specificity of 0.70 will result in a higher number of false positives than true positives. This is a counterintuitive but crucial statistical reality: Even if the test is “good,” its practical value is heavily dependent on the context in which it is applied. Thus, the results may undermine a common assumption surrounding the MMSE: Namely, that despite its limitations in detecting mild cases, a positive result is highly reliable (Mandyla and Kosmidis 2023). Our results challenge this notion: In our study, where the proportion of participants with dementia was around 35%, we already observed a relatively high rate of false positives. In real-world primary care populations, where the prevalence of dementia is expected to be even lower than in our sample, a positive MMSE result would likely produce an even higher rate of false positives, further reducing its diagnostic accuracy. Conversely, although false negatives are fewer in number, their clinical implications are arguably more severe, as missed diagnoses can delay treatment and intervention until it is potentially too late for effective management (Petticrew et al. 2001; Edmonds et al. 2016).

Thus, even under relatively favorable conditions, the MMSE may not provide sufficient diagnostic certainty for primary care clinicians to rely on it exclusively (Mitchell 2012; Arevalo-Rodriguez et al. 2021). Taken together, these findings call for a critical reassessment of the role of the MMSE in primary care cognitive screening. While the test remains a valuable tool, its limitations are often underestimated, particularly in relation to context-dependent variables such as prevalence and population characteristics. Although more comprehensive and nuanced assessments for use in general practice have been proposed (Janssen et al. 2017; Chen et al. 2021), these are not always feasible given the constraints on primary care providers. As a result, clinicians may continue to over-rely on the MMSE despite mounting evidence against its stand-alone utility. To address this gap, future research should focus on developing and validating brief, sensitive, and contextually adaptable tools that GPs can realistically incorporate into everyday clinical workflows. Indeed, improving the early detection of cognitive impairment without increasing the burden on clinicians is not only a methodological challenge but a public health priority (Kasper et al. 2020). Finally, it is important to recognize that, even when more accurate, practical, and brief tools like the Qmci are available, primary care physicians may not use them due to limited awareness or familiarity. Therefore, offering innovative and applicable solutions is not enough, as there must also be adequate training and clear guidelines to support their adoption.

## Limitations and future directions

This study has several limitations that should be acknowledged. First, the diagnosis of dementia was self-reported by clinicians, which limited our control over how diagnoses were established and the specific criteria applied. Second, the study focused broadly on dementia and cognitive impairment without distinguishing between underlying etiologies. While the MMSE is designed to detect cognitive impairment across different conditions, its predictive values may vary depending on the specific type of dementia. Nevertheless, it is important to note that primary care represents the optimal setting for early detection, ideally before progression to severe cognitive impairment or full-blown dementia and, thus, the MMSE may be less suitable than instruments specifically designed to capture milder symptomatology or that, like the Qmci, provide validated and reliable cutoffs for both dementia and MCI. In addition, the present work compared the MMSE to the Qmci within a primary care context, rather than to a wider range of cognitive screening instruments. Future research should, therefore, consider including additional screening tools to enable a more comprehensive evaluation of their relative strengths and to better determine which measures are most effective and feasible for use in primary care settings. Another potential limitation of the present study is that the MMSE and Qmci were administered in a fixed order, as specified in the standardized protocol. Although practice effects are unlikely given the absence of feedback and the minimal overlap between the two instruments (limited to orientation items accounting for a small proportion of the Qmci score), future studies could consider counterbalancing the order of administration to fully exclude possible sequencing effects.

## Conclusions

In conclusion, our findings suggest that even a high-quality test can provide misleading results if used in an inappropriate context. The predictive values of any test are heavily influenced by the a priori prevalence of the condition in the target population, which, in turn, is shaped by the clinical setting. In primary care, this prevalence is typically lower than in specialized clinics, where patients are more likely to experience significant symptoms or be at higher risk.

The goal is not to discourage the use of the MMSE in primary care, but to highlight the importance of understanding its psychometric limitations and avoiding its use as a stand-alone tool. Importantly, the implications of this study extend beyond the MMSE to any cognitive screening instrument. When employing any test, clinicians must be familiar with its psychometric properties and select cutoff scores not only based on general knowledge but also considering the clinical context, individual characteristics (e.g., presence of subjective complaints, family history of neurodegenerative disorders such as Alzheimer’s disease), and the purpose of the assessment (e.g., routine screening vs. symptom-based evaluation).

## Data Availability

Relevant data are provided within the manuscript. The datasets for this article are not publicly available because this study is part of a larger study. Requests to access the datasets should be directed to CP, paul@icbas.up.pt.

## References

[CR1] Arevalo-Rodriguez I, Smailagic N, Roqué-Figuls M et al (2021) Mini-mental state examination (MMSE) for the early detection of dementia in people with mild cognitive impairment (MCI). Cochrane Database Syst Rev. 10.1002/14651858.CD010783.pub3 10.1002/14651858.CD010783.pub3 PMC8406467 34313331

[CR2] Bacsu J, Mateen FJ, Johnson S et al (2020) Improving dementia care among family physicians: from stigma to evidence-informed knowledge. Can Geriatr J 23(4):340 33282053 10.5770/cgj.23.426 PMC7704071

[CR3] Borson S, Small GW, O’Brien Q et al (2023) Understanding barriers to and facilitators of clinician-patient conversations about brain health and cognitive concerns in primary care: a systematic review and practical considerations for the clinician. BMC Prim Care 24:233. 10.1186/s12875-023-02185-4 37932666 10.1186/s12875-023-02185-4 PMC10626639

[CR4] Chen Y-X, Liang N, Li X-L et al (2021) Diagnosis and treatment for mild cognitive impairment: a systematic review of clinical practice guidelines and consensus statements. Front Neurol 12:719849 34712197 10.3389/fneur.2021.719849 PMC8545868

[CR5] Costa E, Pestana J, Barros PP (2024) Primary health care coverage in Portugal: the promise of a general practitioner for all. Hum Resour Health 22:55. 10.1186/s12960-024-00936-7 39123226 10.1186/s12960-024-00936-7 PMC11316367

[CR6] Creavin ST, Wisniewski S, Noel‐Storr AH et al (2016) Mini‐mental state examination (MMSE) for the detection of dementia in clinically unevaluated people aged 65 and over in community and primary care populations. Cochrane Database Syst Rev 10.1002/14651858.CD011145.pub2 PMC8812342 26760674

[CR7] Dos Santos PM, O’Caoimh R, Svendrovski A et al (2019) The rapid community cognitive screening programme (RAPCOG): developing the Portuguese version of the quick mild cognitive impairment (Qmci-P) screen as part of the EIP on AHA twinning scheme. Transl Med UniSa 19:82 31360671 PMC6581493

[CR8] Edmonds EC, Delano-Wood L, Jak AJ et al (2016) missed mild cognitive impairment: high false-negative error rate based on conventional diagnostic criteria. J Alzheimers Dis 52:685–691. 10.3233/JAD-150986 27031477 10.3233/JAD-150986 PMC4879874

[CR9] Fernandes B, Goodarzi Z, Holroyd-Leduc J (2021) Optimizing the diagnosis and management of dementia within primary care: a systematic review of systematic reviews. BMC Fam Pract 22:1–17 34380424 10.1186/s12875-021-01461-5 PMC8359121

[CR10] Fiest KM, Jetté N, Roberts JI et al (2016) The prevalence and incidence of dementia: a systematic review and meta-analysis. Can J Neurol Sci/j Can des Sci Neurol 43(S1):S3–S50. 10.1017/cjn.2016.18 10.1017/cjn.2016.18 27307127

[CR11] Fischer F (2021) Using Bayes theorem to estimate positive and negative predictive values for continuously and ordinally scaled diagnostic tests. Int J Methods Psychiatr Res 30:e1868 33650777 10.1002/mpr.1868 PMC8170576

[CR12] Folstein MF, Folstein SE, McHugh PR (1975) Mini-mental state: a practical method for grading the cognitive state of patients for the clinician. J Psychiatr Res 12:189–198 1202204 10.1016/0022-3956(75)90026-6

[CR13] Janssen J, Koekkoek PS, Moll van Charante EP et al (2017) How to choose the most appropriate cognitive test to evaluate cognitive complaints in primary care. BMC Fam Pract 18:1–8 29246193 10.1186/s12875-017-0675-4 PMC5732477

[CR14] Karimi L, Mahboub-Ahari A, Jahangiry L et al (2022) A systematic review and meta-analysis of studies on screening for mild cognitive impairment in primary healthcare. BMC Psychiatry 22:97. 10.1186/s12888-022-03730-8 35139803 10.1186/s12888-022-03730-8 PMC8827177

[CR15] Kasper S, Bancher C, Eckert A et al (2020) Management of mild cognitive impairment (MCI): the need for national and international guidelines. World J Biol Psychiatry 21:579–594 32019392 10.1080/15622975.2019.1696473

[CR16] Limongi F, Noale M, Bianchetti A et al (2019) The instruments used by the Italian centres for cognitive disorders and dementia to diagnose mild cognitive impairment (MCI). Aging Clin Exp Res 31:101–107. 10.1007/s40520-018-1032-8 30178442 10.1007/s40520-018-1032-8

[CR17] Liss JL, Seleri Assunção S, Cummings J et al (2021) Practical recommendations for timely, accurate diagnosis of symptomatic Alzheimer’s disease (MCI and dementia) in primary care: a review and synthesis. J Intern Med 290:310–334 33458891 10.1111/joim.13244 PMC8359937

[CR18] Mandyla M-A, Kosmidis MH (2023) Limitations and recommendations regarding the Mini-Mental State Examination (MMSE) in illiterate and low educated older adults. Psychol J Hell Psychol Soc 28:141–157

[CR19] Mitchell AJ (2012) The mini-mental state examination (MMSE): an update on its diagnostic validity for cognitive disorders. In: Cognitive screening instruments: a practical approach. Springer, pp 15–46

[CR20] Morgan DJ, Pineles L, Owczarzak J et al (2021) Accuracy of practitioner estimates of probability of diagnosis before and after testing. JAMA Intern Med 181:747–755 33818595 10.1001/jamainternmed.2021.0269 PMC8022260

[CR21] Naugle RI, Kawczak K (1989) Limitations of the mini-mental state examination. Cleve Clin J Med 56:277–281 2743549 10.3949/ccjm.56.3.277

[CR22] O’Caoimh R, Gao Y, Svendrovski A et al (2015) The risk instrument for screening in the community (RISC): a new instrument for predicting risk of adverse outcomes in community dwelling older adults. BMC Geriatr 15:92. 10.1186/s12877-015-0095-z 26224138 10.1186/s12877-015-0095-z PMC4520060

[CR23] O’Caoimh R, William Molloy D (2016) The quick mild cognitive impairment screen (Qmci). In: Cognitive screening instruments: a practical approach. pp 255–272

[CR24] O’Caoimh R, Healy E, Connell EO et al (2012) The community assessment of risk tool (CART): investigation of inter-rater reliability for a new instrument measuring risk of adverse outcomes in community dwelling older adults. In: Irish Journal of medical Science. Springer London LTD

[CR25] Petticrew M, Sowden A, Lister-Sharp D (2001) False-negative results in screening programs: medical, psychological, and other implications. Int J Technol Assess Health Care 17:164–170 11446128 10.1017/s0266462300105021

[CR26] Reisberg B, Ferris SH, De Leon MJ, Crook T (1982) The global deterioration scale for assessment of primary degenerative dementia. Am J Psychiatry 139:1136–1139. 10.1176/ajp.139.9.1136 7114305 10.1176/ajp.139.9.1136

[CR27] Teixeira L, Dos Santos PM, Alves S et al (2017) Screening of dementia in Portuguese primary care: methodology, assessment tools, and main results. Front Med. 10.3389/fmed.2017.00197 10.3389/fmed.2017.00197 PMC5693885 29181378

[CR28] Tran J, Nimojan T, Saripella A et al (2022) Rapid cognitive assessment tools for screening of mild cognitive impairment in the preoperative setting: a systematic review and meta-analysis. J Clin Anesth 78:110682 35193049 10.1016/j.jclinane.2022.110682

[CR29] Villarejo Galende A, Eimil Ortiz M, Llamas Velasco S et al (2021) Report by the spanish foundation of the brain on the social impact of alzheimer disease and other types of dementia. Neurología (English Edition) 36:39–49. 10.1016/j.nrleng.2017.10.004 10.1016/j.nrl.2017.10.005 29249303

[CR30] Webb MPK, Sidebotham D (2020) Bayes’ formula: a powerful but counterintuitive tool for medical decision-making. BJA Educ 20:208–213. 10.1016/j.bjae.2020.03.002 33456952 10.1016/j.bjae.2020.03.002 PMC7808025

[CR31] Wimo A, Seeher K, Cataldi R et al (2023) The worldwide costs of dementia in 2019. Alzheimers Dement 19:2865–2873 36617519 10.1002/alz.12901 PMC10842637

